# Protein Assemblies in Translesion Synthesis

**DOI:** 10.3390/genes15070832

**Published:** 2024-06-24

**Authors:** Gianluca A. Arianna, Dmitry M. Korzhnev

**Affiliations:** Department of Molecular Biology and Biophysics, University of Connecticut Health Center, Farmington, CT 06030, USA; arianna@uchc.edu

**Keywords:** translesion synthesis, DNA damage tolerance, DNA repair, protein structure, protein–protein interactions, protein assemblies

## Abstract

Translesion synthesis (TLS) is a mechanism of DNA damage tolerance utilized by eukaryotic cells to replicate DNA across lesions that impede the high-fidelity replication machinery. In TLS, a series of specialized DNA polymerases are employed, which recognize specific DNA lesions, insert nucleotides across the damage, and extend the distorted primer-template. This allows cells to preserve genetic integrity at the cost of mutations. In humans, TLS enzymes include the Y-family, inserter polymerases, Polη, Polι, Polκ, Rev1, and the B-family extender polymerase Polζ, while in *S. cerevisiae* only Polη, Rev1, and Polζ are present. To bypass DNA lesions, TLS polymerases cooperate, assembling into a complex on the eukaryotic sliding clamp, PCNA, termed the TLS mutasome. The mutasome assembly is contingent on protein–protein interactions (PPIs) between the modular domains and subunits of TLS enzymes, and their interactions with PCNA and DNA. While the structural mechanisms of DNA lesion bypass by the TLS polymerases and PPIs of their individual modules are well understood, the mechanisms by which they cooperate in the context of TLS complexes have remained elusive. This review focuses on structural studies of TLS polymerases and describes the case of TLS holoenzyme assemblies in action emerging from recent high-resolution Cryo-EM studies.

## 1. Introduction

Eukaryotic DNA is continuously altered by endogenous and exogenous genotoxic agents [1,2,3,4,5]. The resulting DNA lesions are removed by DNA repair mechanisms such as base excision repair (BER) [6] or nucleotide excision repair (NER) [7]; however, some damage persists into S-phase, impeding DNA replication. The high-fidelity replicative DNA polymerases, Polδ and Polε, can stall, unable to accommodate structurally aberrant DNA in their restrictive active sites. Replication fork stalling can lead to fork collapse and the build-up of single-stranded (ss) DNA prone to deleterious damage such as double stranded breaks (DSBs) [8,9,10,11]. To protect genomic integrity, cells have evolved DNA damage tolerance (DDT) pathways that enable the replication of damaged DNA without repairing the damage [12,13,14]. The error-prone branch of DDT called translesion synthesis (TLS) employs specialized, low-fidelity DNA polymerases that can take over replication and insert nucleotides across DNA lesions or fill lesion-containing ssDNA gaps left after replication during G2/M-phase [15,16,17,18,19,20,21,22,23,24]. While critical for DNA replication in healthy cells, TLS DNA polymerases are upregulated in many cancers and contribute to cancer resistance to DNA damage induced by genotoxic therapies such as cisplatin [25,26,27,28,29,30,31]. As such, TLS inhibition has emerged as a strategy to enhance the efficacy of first-line chemotherapy in cancer treatment [32,33,34,35,36], highlighting the importance of understanding the structural mechanisms of TLS. Here we review the recent advances in the structural characterization of eukaryotic TLS DNA polymerases and their higher-order assemblies, providing new insights into the concerted action of TLS enzymes during the replicative bypass of DNA lesions.

**Figure 1:**
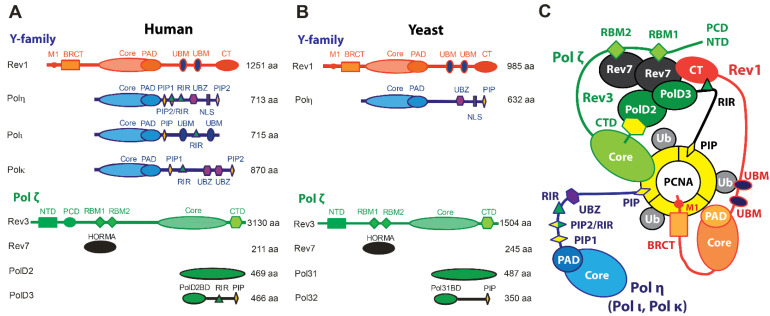
**TLS DNA polymerases in humans and yeast.** (**A**,**B**) Domains and subunits of (**A**) human and (**B**) yeast Y-family polymerases Polη, Polι, Polκ, Rev1, and B-family polymerase Pol ζ. The core catalytic domain of each Y-family polymerase (including fingers, thumb, and palm subdomains) is followed by a polymerase associated domain (PAD). B-family Polζ lacks the PAD domain but includes an inactive endonuclease domain within the catalytic core. The binding modules of Y-family TLS polymerases include PCNA-interacting PIP and Rev1-interacting RIR motifs, ubiquitin-binding UBM (Rev1, Polι) and UBZ (Polη, Polκ) domains, Rev1 N-terminal BRCT and the C-terminal CT domains, and the N-terminal α-helical region of Rev1 termed the “M1” motif. Polη includes a nuclear localization signal (NLS). Polζ comprises four subunits: Rev3, Rev7, PolD2/PolD3 (humans), or Pol31/Pol32 (yeast). The Rev3 subunit of Polζ harbors a catalytic core domain, an N-terminal domain (NTD), a positively charged domain (PCD, humans), two Rev7-binding motifs (RBMs), and a C-terminal domain (CTD) interacting with the PolD2/PolD3 or Pol31/Pol32 module. (**C**) Schematic of Rev1/Polζ-dependent TLS mutasome assembled on ubiquitinated PCNA, including an ‘inserter’ TLS polymerase Polη, and ‘extender’ TLS polymerase Polζ and a scaffold protein Rev1. Possible interactions between domains and subunits of the TLS proteins are indicated. Adapted with permission from [32].

In humans (*Hs*), enzymes of the TLS pathway include the Y-family DNA polymerases Polη, Polι Polκ, Rev1, and B-family Polζ, while *S. cerevisiae* (*Sc*) or baker’s yeast, which has long served as a model organism for studying TLS in eukaryotes, possesses Polη, Rev1, and Polζ only [15,16,17,18,19,20,21,22,23,24] (Figure 1A,B). Unlike Y-family DNA polymerases that comprise a single polypeptide chain, Polζ is a heteroprotein complex of the catalytic subunit Rev3, the two copies of HORMA domain subunit Rev7, and two accessory subunits, *Hs* PolD2/PolD3 or *Sc* Pol31/Pol32, that are also subunits of the replicative B-family DNA polymerase Polδ [37,38,39,40,41] (Figure 1). Beyond their catalytic domains, TLS polymerases feature accessory motifs, domains, and subunits that mediate protein–protein interactions (PPIs) involved in TLS regulation (Figure 1A,B) that control TLS polymerase recruitment and assembly into multiprotein complexes at sites of DNA of damage.

The catalytic domains of TLS DNA polymerases generally (i) have active sites that are adapted to accommodate DNA lesions, (ii) form overall fewer contacts with primer/template (p/t) DNA than the replicative polymerases, and (iii) are more prone to incorrect base pairing due to the lack of 3′-5′ exonuclease activity [23,42]. These properties provide TLS enzymes with the ability to replicate past sites of DNA damage at the expense of decreased replication fidelity, processivity, and efficiency on undamaged DNA [15,16,17,18,19,20,21,22,23,24]. While the nucleotide misincorporation rates of the replicative DNA polymerases Polδ and Polε are ~10^–6^–10^–8^, the misincorporation rates of the TLS DNA polymerases range from 10^–1^ to 10^–4^ [43,44].

The access of mutagenic TLS enzymes to DNA is regulated through PPIs with proliferating cell nuclear antigen (PCNA), a homo-trimeric sliding clamp that serves as a polymerase processivity factor and a scaffold for DNA replication and damage response proteins [45,46,47,48] (Figure 1C). Like other PCNA binding partners, Polη, Polι, Polκ, and the *Hs* PolD3/*Sc* Pol32 subunit of Polζ contain PCNA-interacting protein box (PIP-box) motifs that mediate recruitment of TLS polymerases to DNA damage-induced foci [49,50,51,52,53,54,55,56] (Figure 1A,B). In contrast, Rev1 lacks a PIP-box motif and instead binds PCNA via its BRCA1 C-terminus (BRCT) domain [57,58], which is preceded by an α-helical M1 region that reportedly interacts with the recessed 5′ phosphorylated terminus of ssDNA [59] (Figure 1A,B). The switch from high-fidelity replication to TLS is prompted by the mono-ubiquitination of PCNA at residue K164 by the Rad6 ubiquitin-conjugating enzyme (E2) and Rad18 ubiquitin-protein ligase (E3) pair, which is recruited to ssDNA coated with replication protein A (RPA) formed at stalled replication forks [60,61,62,63,64,65]. This post-translational modification enhances the affinity of PCNA for the Y-family TLS DNA polymerases, which bind a PCNA-attached ubiquitin moiety via ubiquitin-binding motif (UBM) or ubiquitin binding zinc-finger (UBZ) domains [66,67] (Figure 1A,B).

Individual TLS DNA polymerases can copy over certain DNA lesions, as exemplified by the accurate and efficient bypass of UV-induced T-T cyclobutane pyrimidine dimers (CPDs) by Polη [68]. However, the replicative bypass of most types of DNA damage generally requires a two-step *Rev1/Polζ-dependent TLS* [69,70,71,72], which includes (i) base insertion across from the lesion by Polη, Polι, or Polκ, and (ii) the extension of the structurally aberrant primer-template, typically handled by Polζ [69,70,71,72]. This process involves the coordinated action of several TLS DNA polymerases assembled into a complex called *the TLS mutasome* whereby PCNA and Rev1 serve as scaffolds (Figure 1C). Rev1 plays a key structural role in Rev1/Polζ-dependent TLS by coordinating polymerase recruitment, selection, and switching via PPIs of its C-terminal domain (Rev1-CT), while its limited catalytic activity as dCMP transferase is dispensable for TLS [73,74,75,76,77]. The Rev1-CT domain features two independent PPI interfaces, one for the Rev7 subunit of Polζ and one for Rev1-interacting regions (RIRs) of *Hs* Polη, Polι, Polκ, and the PolD3 subunit of Polζ that are missing in TLS proteins in yeast (Figure 1) [78,79,80,81,82,83,84,85].

The architecture of Polζ, which serves as a master ‘extender’ polymerase within the TLS mutasome [37], resembles that of other multi-subunit B-family enzymes such as the primase Polα or the replicative polymerases Polδ and Polε [24,86,87,88,89]. The Rev3 subunit of Polζ includes the catalytic domain followed by a cysteine-rich C-terminal domain (Rev3-CTD) that binds Zn and [4Fe-4S] cluster [90] via CysA and CysB motifs, respectively (Figure 1A,B) [37,38,39,40,41]. Rev3-CTD aligns with the corresponding region of Polδ harboring the [4Fe-4S] cluster [90], and mediates PPIs with the *Hs* PolD2/PolD3/*Sc* Pol31/Pol32 module shared between Polδ and Polζ [37,38,39,40,41]. The Rev3 subunit also features a long insert between its N-terminal domain (NTD) and an inactive 3′-5′ exonuclease domain [17,91] containing a pair of Rev7-binding motifs (RBMs) that bind two copies of the accessory subunit Rev7 [92,93,94] (Figure 1A,B). Both *Hs* and *Sc* Rev7 can form homodimers within Polζ, although the dimerization mode is reported to be different in each case [94,95]. The accessory Rev7 and *Hs* PolD2/PolD3/*Sc* Pol31/Pol32 subunits are important for Polζ function, significantly enhancing its catalytic efficiency [38,40,41].

This review focuses on structural studies underpinning the mechanisms by which TLS polymerases catalyze replication across DNA lesions in the context of multiprotein complexes. Following a brief overview of the key proteins of the TLS pathway provided in the introduction, we will discuss the structural basis of DNA synthesis by catalytic domains of Y-family Polη, Polι, Polκ, Rev1, and B-family Polζ revealed by X-ray crystallography studies (reviewed in [21,22,23,24]) and recent cryo-EM structures of the *Sc* Polζ holoenzyme [96,97,98,99,100]. While much is known about TLS PPIs, the majority of their structural studies have focused on individual domain–domain or domain–peptide interactions. Given the number of possible PPIs within the TLS mutasome, it represents a multivalent complex that adopts different configurations at each step of the lesion bypass, allowing for effective polymerase recruitment, selection, and switching. A structural picture of the full TLS mutasome in the act of nucleotide insertion across from the lesion or p/t DNA extension has yet to be realized. Hoverer, the recent landmark Cryo-EM structures of TLS holoenzyme complexes, including the structure of *Hs* Polκ in association with PCNA and DNA [96], the structures of *Sc* Polζ on and off p/t DNA [97,98,99], and the partial structure of the Rev1/Polζ complex during p/t extension [100], provide new mechanistic insights into the TLS DNA polymerase assemblies. Herein, we will overview the key findings of these works along with the previous structural studies of PPIs within the TLS mutasome, and discuss their implications for TLS mechanisms.

## 2. Structural Basis of DNA Synthesis by TLS Polymerases

### 2.1. Catalytic Domains of the Y-Family TLS Polymerases

**Figure 2:**
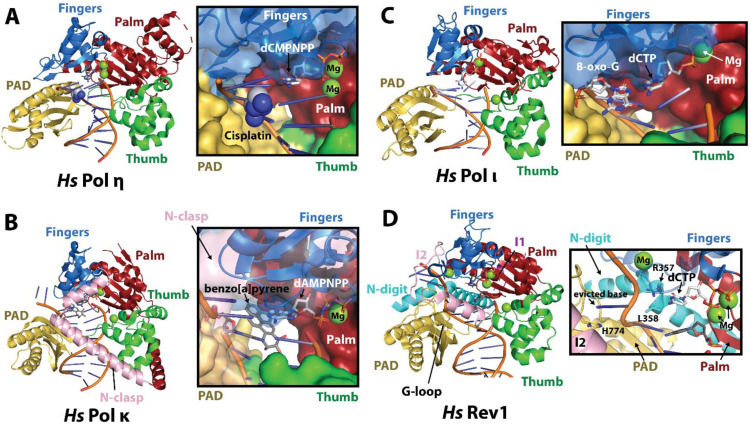
**Catalytic domains of human Y-family TLS DNA polymerases**. Each Y-family polymerase features fingers (blue), palm (red), thumb (green), and PAD (yellow) domains. Insets for A–D show close-ups of polymerase active sites in the process of nucleotide insertion. (**A**) Hs Polη bypassing a cisplatin DNA adduct. (**B**) Polκ bypassing benzo[a]pyrene adduct. (**C**) Hs Polι bypassing 8-oxoG lesion. (**D**) Hs Rev1 inserting cytosine on a normal p/t DNA.

Crystal structures of the catalytic cores of the Y-family TLS DNA polymerases have been determined with and without unmodified or damage-containing DNA substrates (reviewed in [21,22,23,24]). These structures have unveiled the mechanisms by which TLS DNA polymerases recognize, accommodate, and insert bases across various DNA lesions. The catalytic regions of Polη, Polι, Polκ, and Rev1 consist of finger, palm, and thumb domains common to all DNA polymerase families, and a C-terminal ‘little finger’ (LF) or polymerase associated domain (PAD), which is unique to Y-family polymerases (Figure 2A–D) [15,16,17,18,19,20,21,22,23,24]. The catalytic core structure resembles a right hand holding p/t DNA between the thumb and little finger domains positioning it for incoming dNTP. As with all DNA polymerase families, the catalytic site in the palm domain contains carboxylate residues that coordinate three Mg^2+^ ions that are needed to catalyze phosphodiester bond formation between the 3′-OH of the primer strand and α-phosphate of the incoming nucleotide [23,101,102]. Contributing to the ability to accommodate aberrant DNA, the finger and thumb domains of the Y-family enzymes are shorter and form fewer interactions with DNA compared to the replicative B-family polymerases, while the PAD domain serves to stabilize the templating base through contacts with the DNA backbone. Another contributing factor to the proficiency of the Y-family polymerases to incorporate nucleotides over DNA lesions is a lack of the 3′-5′ exonuclease domain and thus proofreading capability, which greatly decreases replication fidelity on undamaged DNA. It has also been noted that, unlike replicative polymerases, some Y-family polymerases (such as *Hs* Polη) preexist in a ready-for-catalysis state and undergo little conformational changes induced by nucleotide binding [103,104].

Like B-family replicative polymerases, the Y-family TLS polymerases feature steric gating residues within their active sites that allow for sugar discrimination during catalysis. By this mechanism, a bulky residue within the active site of a B- or Y-family DNA polymerase (typically Tyr or Phe) clashes with the 2′-OH of an incoming rNTP, averting ribonucleotide incorporation via steric exclusion [105,106,107,108,109,110]. *Hs* Rev1 is unique in that it utilizes a steric gate in combination with a polar filter residue, which interacts with the 3′-OH and brings the primer nucleotide closer to the steric gate [111]. In general, the incorporation efficiency of rNTP by TLS DNA polymerases is lower relative to dNTP; however, it varies across the different enzymes. For example, *Sc* Polη and Polζ are fairly intolerant of rNTPs and do not incorporate them frequently [109,112,113,114,115]. In contrast, *Hs* Polη incorporates dNTP and rNTPs with similar efficiency across 8-oxo-G and TT CPD, while *Hs* Polι was reported to incorporate rNTPs across abasic sites [109,112,116,117].

Despite having similar architecture, the catalytic cores of Y-family TLS polymerases have unique structural features and vary in interactions with DNA. For example, the catalytic region of Polκ includes an N-terminal extension (N-clasp) that links the core and PAD domains, stabilizing the latter on DNA (Figure 2B) [118]. In a similar manner, the core and PAD domains of Rev1 are not contiguous but held together by an N-terminal extension (N-digit), which makes further contacts with the DNA minor groove (Figure 2D) [119,120,121]. These structural details determine the types of DNA damage (cognate lesions) that each Y-family polymerase can recognize and accurately bypass [23].

*Polη* specializes in the bypass of cyclobutane pyrimidine dimers (CPDs) [68], which are the most common lesion induced by UV light, and interstrand crosslinks induced by platinum-based chemotherapies, such as cisplatin [122]. Crystal structures of the *Hs* Polη in complex with DNA containing a TT CPD [123] or platinum cross-linked guanines (GpG) (Figure 2A) [30,124] reveal that the active site of Polη constrains DNA in B-form, minimizing the distortions caused by the lesion and thus stabilizing Watson–Crick (WC) geometry with the correct incoming nucleotide. While Polη can insert dATP across TT CPD and dCTP across GpG in an error-free manner, it tends to misincorporate dGTP across T and has an error rate of 10^−2^–10^−3^ on undamaged DNA [125].

*Polκ* is particularly adept at the accurate bypass of DNA lesions induced by bulky polycyclic aromatic hydrocarbons (PAHs), such as benzo[a]pyrene guanine adducts (BPdG) [23]. The crystal structure of Polκ in complex with p/t DNA containing a BPdG lesion reveals an open active site adapted to accommodate the bulky minor groove adduct (Figure 2B) [126]. This property stems from the loose association between the catalytic core and PAD domains bridged by the N-clasp, which stabilizes Polκ on aberrant p/t DNA allowing it to insert the correct dCTP nucleotide. Following the nucleotide insertion, the BPdG adduct in the 5′ position translocates down one position and orients towards the DNA minor groove. Not only is this conformation energetically favorable, but the lesion also sterically clashes with mismatches in the nascent base pair, thus favoring correct pairing and allowing Polκ to extend past the lesion with reasonable accuracy.

*Polι*, unlike Polη and Polκ, has a much narrower active site, which binds and constrains template bases in a *syn* conformation and favors Hoogsteen over WC base pairing [23]. The constraints placed by its active site make Polι accurate across template purines (dA, dG), since favorable hydrogen bonds to dTTP and dCTP in an *anti* conformation are still formed along the Hoogsteen edge. However, this also makes Polι much more promiscuous when inserting dNTPs across template pyrimidines (dC, dT). Crystal structures of *Hs* Pol ι in complex with an 8-oxo-guanine (8-oxoG) lesion demonstrate how this property aids Polι in correctly incorporating dCTP across this lesion (Figure 2C) [127]. The 8-oxoG is stabilized in the *syn* conformation and forms optimal hydrogen bonds to dCTP in the *anti* conformation, thus promoting accurate lesion bypass.

*Rev1* is unique among Y-family TLS polymerases in that it primarily functions as a dCMP transferase, preferentially inserting cytosine regardless of the template base [23,73]. Crystal structures of *Hs* Rev1 on undamaged DNA suggest this property stems from a protein templating mechanism (Figure 2D) [111,119]. The residue L358 on the N-digit of *Hs* Rev1 evicts the template base, orienting it away from the DNA helix and stabilizing it through interactions with H774 in the PAD domain. *Hs* Rev1 then uses R357 (R324 in *Sc* Rev1) on the N-digit as the new ‘template’, which forms WC-like hydrogen bonds with the incoming dCTP. This unusual mechanism enables Rev1 to bypass lesions such as adducted guanines or abasic sites.

### 2.2. Catalytic Domain of the B-Family TLS Polymerase Polζ

The catalytic domain of Polζ harbored in the Rev3 subunit has proven a particular challenge for structural characterization. Historically, due to its large size (3103 and 1505 amino acids for *Hs* and *Sc* Rev3) and tendency toward aggregation, the purification of Rev3 alone has been difficult, hindering efforts to obtain high-resolution structures [41,128]. In 2013, Gómez-Llorente et al. reported the first low-resolution EM structure of the multisubunit *Sc* Polζ complex at 22 Å resolution [128]. In 2020, Malik et al. presented breakthrough high-resolution Cryo-EM structures of the *Sc* Polζ holoenzyme on (3.1 Å) and off (4.1 Å) undamaged p/t DNA [97], followed by a structure of Polζ with a base pair mismatch at the primer terminus (3.1 Å) [98]. These studies not only uncovered the architecture of the heteropentameric *Sc* Polζ assembly, consisting of Rev3, Pol31, Pol32, and two copies of Rev7 (Rev7_A_ and Rev7_B_) subunits, but also provided structural insights into the function of Polζ as a master extender polymerase during Rev1/Polζ-dependent TLS.

**Figure 3:**
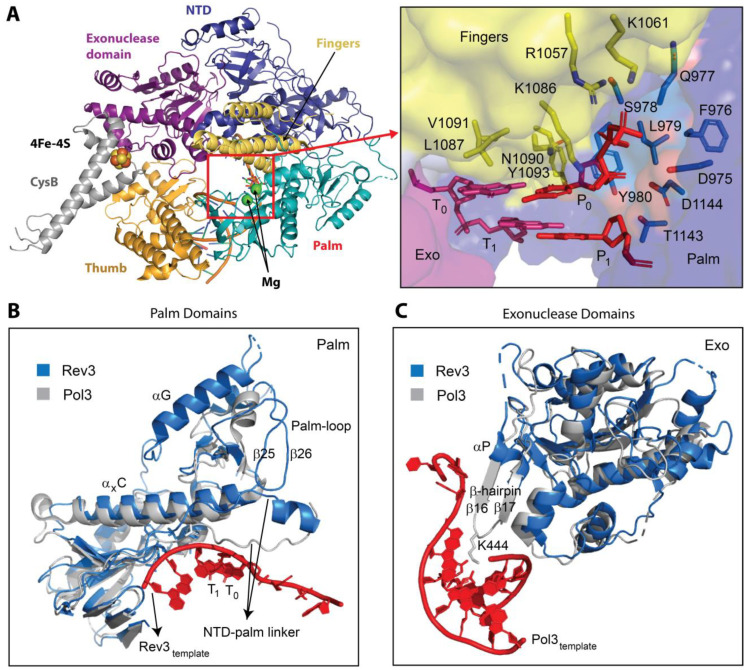
**Cryo-EM structure of the Sc Polζ catalytic domain**. (**A**) The Sc Rev3 catalytic domain on p/t DNA, including the fingers (yellow), thumb (orange), palm (cyan), NTD (blue), and inactive exonuclease (purple) domains [97]. The CysB domain within Rev3 CTD that binds Pol31 is also shown. The inset shows a close-up of the Rev3 active site, revealing the basis for Polζ intolerance to mismatches during nucleotide insertion. Residues from the fingers (yellow) and palm (blue) domains make extensive contacts with the nascent base pair (red). (**B**) Structural basis for Polζ extension past DNA mismatches [97]. Due to contacts with α_x_C and palm-loop, the NTD-palm linker in the Rev3 active site is held further away from DNA than in Pol3. (**C**) Structural basis of the impaired exonuclease activity of Polζ [97]. Unlike Pol3, Rev3 lacks the β-hairpin used to pass mismatched DNA from the active site to the exonuclease domain.

The catalytic core of *Sc* Polζ [97] bears resemblance to that of replicative B-family polymerases Polδ and Polε [24,86,87,88,89], featuring a palm domain that carries catalytic carboxylate residues, a finger domain that makes contacts to the nascent DNA pair, a thumb domain that interacts with the minor groove of DNA, an exonuclease domain which is inactive in Polζ, and an N-terminal domain (NTD) that bridges exonuclease and fingers domains (Figure 3A). The thumb, palm, and fingers of Rev3 grasp DNA tightly, creating an active site that is fairly intolerant to mismatches. Like high-fidelity replicative polymerases, Rev3 makes contacts to the nascent base pair via residues in its fingers and palm domain that enforce WC geometry at the templating position T_0_P_0_ (here T is template, P is primer; the subscript denotes the number of bases past the templating position) (Figure 3A, inset). However, Rev3 can tolerate mismatches and DNA lesions at the T_1_P_1_ position, which sets Polζ apart from the replicative polymerases and contributes to its extender function in TLS. While in the Pol3 subunit of Polδ, an NTD-palm linker spans along p/t and forms contacts constraining the T_1_ nucleotide; the NTD-palm linker in Rev3 moves away from the template, creating a cleft near the T_1_ position that can accommodate structurally aberrant DNA (Figure 3B). Another key feature that enables Polζ to extend past DNA lesions is an inactive Rev3 exonuclease domain, which lacks the carboxylate residues found in other B-family polymerases that mediate 3′-5′ exonuclease activity and misses a crucial β-hairpin that regulates the transition of mismatched p/t from the active site to the exonuclease domain for proofreading (Figure 3C).

In 2022, Malik et al. reported another Cryo-EM structure of the *Sc* Polζ holoenzyme, this time in complex with a mismatched DNA primer-template [98]. While the overall architecture of Polζ is preserved in this structure, the fingers domain adopts a more open conformation and, based on a less defined EM density, becomes more dynamic, loosening the enzyme’s grip on DNA and allowing it to accommodate the mismatch. Despite a reduced number of contacts with the active site, the nascent base pair is still constrained in WC geometry by some interactions with the fingers domain, revealing how Polζ may catalyze DNA synthesis past the mismatch. Taken together, the structures of *Sc* Polζ on matched and mismatched p/t DNA have unmasked the structural determinants of Polζ function during the extension step of TLS.

## 3. Protein Interaction Modules—The Building Blocks of TLS Complexes

Catalytic domains of TLS polymerases furnish an enzymatic toolbox for the replicative bypass of DNA lesions. In addition, the recruitment of TLS polymerases to DNA, selection of polymerase(s) appropriate for the type of damage, and polymerase switching are governed by a network of PPIs mediated by accessory motifs, domains, and subunits of the TLS enzymes (Figure 1). Structural studies of individual PPIs within this network mainly focused on the TLS scaffold proteins, PCNA and Rev1, and the Rev7 subunit of Polζ. These works portrayed the building blocks of the multiprotein TLS complexes and provided insights into TLS regulation.

### 3.1. Interactions with PCNA

**Figure 4:**
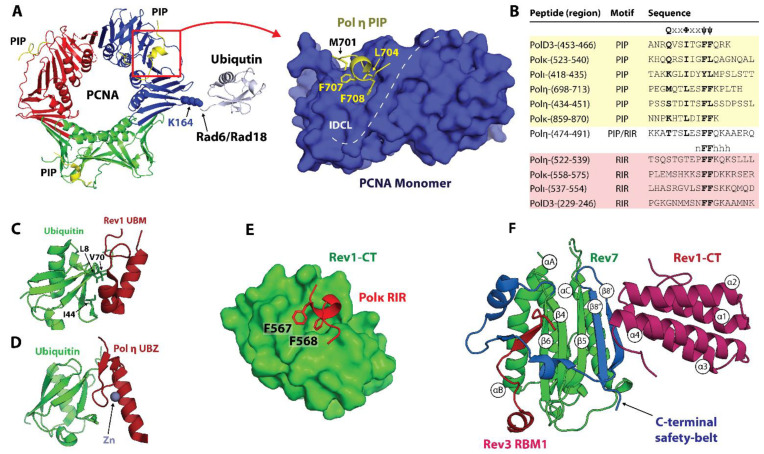
**Protein-protein interactions in TLS**. (**A**) Hs PCNA with a Polη PIP-box peptide bound near the interdomain connector loop (IDCL) [130]. Ubiquitin (gray) is ligated at K164 of PCNA by the Rad6/Rad18 E2/E3 pair. A close-up view of the PCNA monomer showing a “three-pronged fork” of Polη PIP (L704, F707, F708) inserting into a hydrophobic pocket in the IDCL region of PCNA, and M701 binding to the Q-pocket. (**B**) PIP-box and RIR sequences in the human TLS polymerases aligned to their minimal motifs. (**C**) Hs Rev1 UBM2 bound to a conserved site on ubiquitin centered at L8, I44, and V70 [135]. (**D**) A model of Hs Polη UBZ bound to ubiquitin (based on the Rad18-UBZ/ubiquitin complex) [132,137]. (**E**) Hs Polκ RIR motif bound to Rev1-CT with F567 and F568 inserted in a hydrophobic pocket in the N-terminal part of the domain [85]. (**F**) Ternary complex of Hs Rev7, Rev3 RBM1, and Rev1-CT [83]. Rev7 uses a C-terminal ‘safety-belt’ loop (blue) to latch the Rev3 RBM1 peptide to the core. The C-terminus of the ‘safety-belt’ region forms a β8′-β8′’ hairpin, which extends the core β-sheet (β4-6) and provides the binding interface for Rev1-CT.

The sliding clamp PCNA is a polymerase processivity factor and an interaction hub that orchestrates protein recruitment and transactions taking place on DNA during replication and repair [45,49,50]. Eukaryotic PCNA is a ring-shaped trimer of identical subunits that encircles DNA, with each subunit consisting of the two domains linked by the interdomain connector loop (IDCL) (Figure 4A). Most PCNA partners harbor one or several PCNA-interacting protein box (PIP-box) motifs with the consensus sequence **Q**_1_x_2_x_3_**φ**_4_x_5_x_6_**ψ**_7_**ψ**_8_ (where φ is I/L/M/V, ψ is F/Y, x is any residue), which bind a conserved site in the IDCL region of PCNA [49,50,51,52,53,54,55,56] (Figure 4A,B). Upon binding, the PIP-box forms a helix that orients residues in positions 4, 7, and 8 (the ‘three-pronged plug’) towards a hydrophobic pocket on PCNA, while Q in position 1 inserts into a site termed the ‘Q-pocket’. For the tightest-binding PIP-box motifs such as the one from p21^WAF1/CIP1^ (*K_d_* for *Hs* PCNA of 80 nM [50]), the PPI is enhanced by residues C-terminal to the consensus sequence that forms an intermolecular β-sheet with the IDCL of PCNA and/or other peripheral contacts [50,129].

The Y-family TLS polymerases Polη, Polι, and Polκ bind PCNA using non-canonical PIP-box motifs that deviate from the consensus sequence [51,52,53,54,55,56,130] (Figure 1 and Figure 4B). In contrast, the B-family TLS polymerase Polζ borrows a canonical PIP-box from the *Hs* PolD3/*Sc* Pol32 subunit shared with the replicative DNA polymerase Polδ (Figure 1). Unlike other Y-family polymerases, Rev1 lacks a PIP-box and binds PCNA via its N-terminal BRCT domain (Figure 1) [57,58]. Crystal structures of *Hs* PCNA in complex with PIP-box peptides derived from *Hs* Polη, Polι, and Polκ reveal that these non-canonical motifs bind the IDCL region of PCNA using the ‘three-pronged fork’ mechanism, while the residue in position 1 lacks (Polι, Polκ) or forms suboptimal (*Hs* Polη) interactions with the ‘Q-pocket’ (Figure 4A, left) [130]. The overall fewer contacts with PCNA lead to weaker PCNA binding affinities for non-canonical sequences (*K_d_* in the μM range) relative to the canonical PIP-boxes [50,129,130]. The relatively weak PPIs of non-canonical PIP-boxes with PCNA promote TLS polymerase recruitment to DNA [51,52,53,54,55,56], while also permitting the selection and switching of the TLS polymerases as they compete for space on the sliding clamp.

In addition to PIP-box motifs, all Y-family TLS polymerases have ubiquitin-binding UBZ and UBM domains lacking in replicative polymerases (Figure 1). Rad6/Rad18-dependent mono-ubiquitination of PCNA at sites of DNA damage leads to UBM and UBZ binding to PCNA-attached ubiquitin moieties and promotes the replacement of replicative with TLS polymerases by an affinity-driven competition [66,67]. Polη and Polκ harbor one and two UBZ domains, respectively (Figure 1 and Figure 4D), while Polι and Rev1 each feature two UBM domains (Figure 1 and Figure 4C). Solution NMR studies reveal that the *Hs* Polη UBZ domain adopts a canonical ββα type-III C_2_H_2_ zinc-finger fold coordinating a single Zn^2+^ ion [131,132], while *Hs* Rev1 and Polι UBM domains have an αα structure [133,134,135,136]. Both UBZ and UBM domains engage a conserved site on ubiquitin centered at L8, I44, and V70 (Figure 4E), although mutation of I44 is not sufficient to disrupt the binding of the Rev1 UBM2 domain [135]. Notably, only one of the two UBMs in Rev1 (UBM2) was shown to interact with ubiquitin in both humans and yeast [135].

### 3.2. Scaffolding Function of Rev1

The two unique modular domains in Rev1 that are not found in other Y-family polymerases, Rev1-BRCT and Rev1-CT (Figure 1), enable its function as a central scaffold protein of the TLS pathway [57,74,77,82,84]. In 1971, the *rev1-1* strain of yeast was discovered, which contains the G193R mutation in Rev1, displaying a DNA damage-sensitive phenotype and a marked decrease in mutagenesis [138]. This mutation maps onto the BRCT region of *Sc* Rev1 and has no effect on catalysis, which has led to the suggestion of a ‘second’ structural role of Rev1 in TLS [74]. The *Hs* Rev1-BRCT domain promotes resistance to DNA damage and mediates *Hs* Rev1 localization to replication foci [57,139]. While the structures of both *Hs* and *Sc* Rev1-BRCT have been determined [58,140], the interactions of this domain have long remained unknown, with tentative partners including PCNA [57,58], DNA [59], and phosphopeptides [141]. The direct interaction of *Sc* Rev1-BRCT and PCNA with *K_d_* of ~79 μM was demonstrated by NMR, revealing that the BRCT domain competes with PIP-box motifs for the same site in the IDCL region of PCNA [58]. The precise structural mechanism by which PCNA can accommodate Rev1-BRCT in a binding site reserved for other partners remains to be established.

The Rev1-CT domain is a versatile interaction module that mediates Rev1 PPIs with other Y-family TLS polymerases and the accessory subunits of the B-family TLS polymerase Polζ [57,74,77,82,84]. The two independent interfaces of *Hs* Rev1-CT can simultaneously bind the Rev7 subunit of Polζ and the Rev1-interacting regions (RIRs) of *Hs* Polη, Polι, and Polκ (Figure 1 and Figure 4B) [78,79,80,81,82,83,84,85], bridging the extender and inserter polymerases during Rev1/Polζ-dependent TLS. In addition, an RIR motif has been identified in the *Hs* PolD3 subunit of Polζ, which is implicated in an inserter-to-extender polymerase switch (Figure 1 and Figure 4B) [82]. The RIR motif has a consensus sequence of n**FF**hhhh, where h is a helix-forming residue, and n is an N-capping residue [142] (Figure 4B) [78,80]. Solution NMR structures of Rev1-CT bound to RIR peptides from *Hs* Polη [80] and PolD3 [82] reveal that, upon binding, the RIR motif folds into an α-helix, directing the FF pair towards a pocket in the N-terminal part of Rev1-CT (Figure 4E). Crystal structures of the Rev1-CT/Rev7 complexes show that Rev7 binds to the opposite C-terminal face of Rev1-CT without obstructing the RIR interface (Figure 4F) [83,84,85]. The overlapping PIP-box and RIR motifs were identified in human TLS polymerases sharing a pair of aromatic residues (Figure 4B), challenging the notion of PIP and RIR as distinct entities [143]. Notably, the TLS polymerases in yeast lack a putative RIR motif, although *Sc* Rev1-CT can bind an R**FF**D sequence from *Sc* Rad5 in a manner similar to *Hs* Rev1-CT/RIR PPI [144]. It has been proposed, therefore, that *Sc* Polη utilizes its C-terminal PIP-box motif for Rev1-CT binding [145].

### 3.3. Interactions of Polζ Subunits

Previous structural studies of Polζ mainly focused on interactions of its accessory Rev7 subunit with the Rev7-binding motifs (RBMs) from Rev3 [95,146] and with the Rev1-CT domain [83,84,85], although structures of the *Hs* PolD2/PolD3 and *Sc* Pol31/Pol32 accessory modules are also available [86,147]. The two consecutive RBMs were identified in both *Hs* and *Sc* Rev3 located in the long insert between its NTD and exonuclease domains (Figure 1) [92,93,94]. In humans, the core RBM sequence is defined as ΦΦψ**P**xxxp**P**, where Φ is an aromatic or bulky aliphatic residue, ψ is a residue with a long aliphatic side-chain, n is an N-capping residue [142], while in yeast the first conserved P may be replaced with Y [94]. Rev7 interacts with RBMs using a mechanism unique to HORMA proteins whereby the Rev7 ‘safety-belt’ loop closes on the top of the ligand, securing it to the protein core by the β8′-β8′′ hairpin, which extends the Rev7 β-sheet (Figure 4F) [95,146]. This newly formed β-hairpin creates a binding site of Rev7 for the Rev1-CT domain [83,84,85]. Within Polζ, the two sequential RBMs of Rev3 recruit two copies of Rev7 [94,95]. The two *Hs* Rev7 protomers tethered by the Rev3 fragment form a symmetric head-to-head dimer via the canonical HORMA interface centered at helix αC [95], while the two copies of *Sc* Rev7 are forced into an unusual head-to-head dimer in the *Sc* Polζ assembly [94,98]. The Rev3/Rev7 complex formation results in a ~20–30 fold increase in the Rev3 catalytic activity [148], which is further improved by the addition of the *Hs* PolD2/PolD3 or *Sc* Pol31/Pol32 block [38,41]. The loss of *Hs* Rev7 PPIs with Rev3-RBMs [92] or *Hs* Rev7 dimerization [95] sensitizes cells to DNA damage induced by UV and cisplatin, highlighting the importance of Rev7 as a key interaction module of the Polζ assembly.

## 4. Molecular Assemblies in TLS

**Figure 5:**
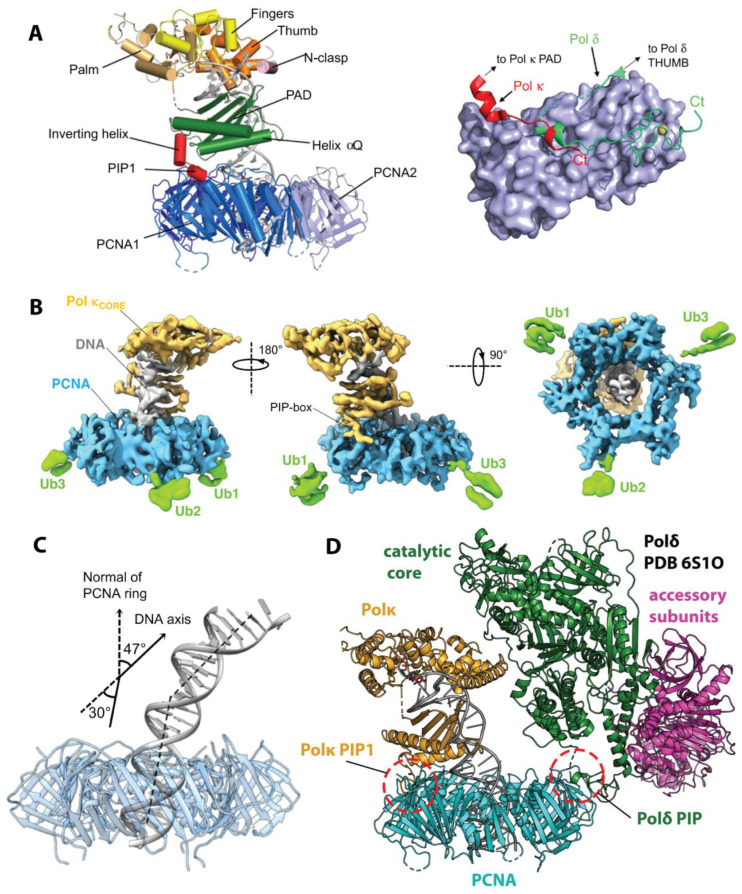
**Cryo-EM structure of the human Polκ holoenzyme**. (**A**) Polκ bound to p/t DNA (gray) and the front face of unmodified PCNA (blue). The catalytic core of Polκ comprises the palm (tan), fingers (yellow), thumb (orange), N-clasp (pink), and PAD domains (green). An ‘inverting’ helix C-terminal to PAD orients the internal Polκ PIP-box motif (red) towards the binding site on the PCNA1 protomer. The right panel shows a structural comparison of the Hs Polκ PIP1 and Hs Polδ PIP motifs bound to a PCNA protomer. (**B**) Polκ in complex with p/t DNA and mono-ubiquitinated PCNA with ubiquitin moieties visible on the PCNA back face. (**C**) DNA that exits the Polκ holoenzyme tilts 47° relative to the PCNA axis, resulting in DNA bending when passing through the PCNA ring. (**D**) A model of a PCNA ‘tool belt’ that simultaneously binds to the Polκ/DNA (yellow/gray) complex and apo Polδ (green/magenta). Adapted with permission from [96].

**Figure 6:**
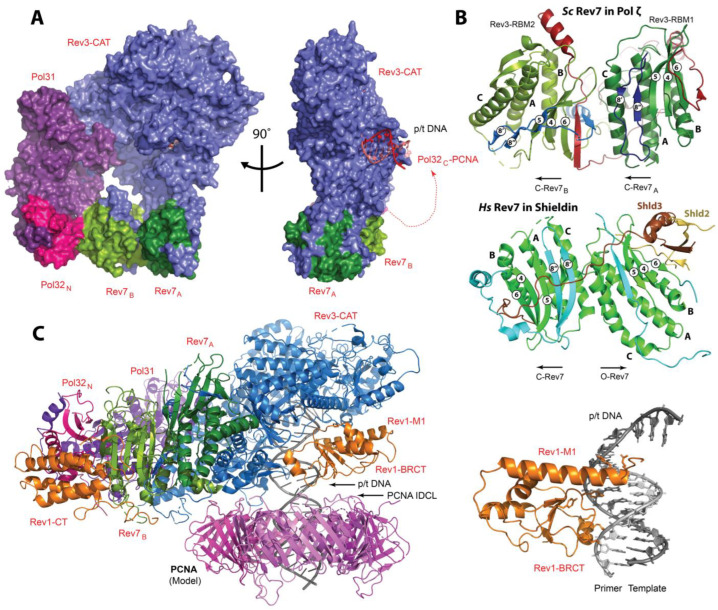
**Cryo-EM structures of the *Sc* Polζ and *Sc* Rev1/Polζ complex**. (**A**) “Daisy-chain” or ring-like architecture of the five-subunit *Sc* Polζ holoenzyme on p/t DNA. Rev3 accommodates p/t DNA (red) and makes contacts to Pol31, Rev7_A_, and Rev7_B_ but not Pol32 [97]. Rotation of the structure shows an unstructured C-terminus of Pol32, which contains a PIP-box motif, reaching a tentative PCNA location. (**B**) An unusual head-to-tail *Sc* Rev7 dimer within Polζ (**top**) that does not utilize the canonical HORMA dimerization interface [97]. For comparison, a head-to-head dimer is shown (**bottom**) formed by *Hs* Rev7 within the shielding complex [149]. Here the heterodimer of closed (C-Rev7) and open (O-Rev7) protomers is formed via the interface centered at helix αC. (**C**) Partial structure of the *Sc* Rev1/Polζ complex in which the *Sc* Polζ holoenzyme is engaged with p/t DNA [100]. Only the Rev1 CT, BRCT, and M1 regions are visible. The PCNA ring is modeled based on the structure of Polδ/PCNA complex [87]. A close-up view of the Rev1-M1 and Rev1-BRCT interactions with p/t DNA is shown on the right [100].

The recent advent of high-resolution Cryo-EM has opened an avenue for the structural characterization of multiprotein assemblies acting in TLS. Following the high-resolution structures of the multisubunit Polδ and Polε holoenzymes [86,87,88,89], structures have been determined for TLS complexes, including full-length *Hs* Polκ bound to PCNA and p/t DNA [96] (Figure 5), the *Sc* Polζ holoenzyme on and off matched and unmatched p/t DNA [97,98,99], and the Rev1/Polζ complex during p/t extension [100] (Figure 6). These structures captured TLS machines in action, piecing together the catalytic and regulatory modules and unveiling how TLS enzymes cooperate during replication and lesion bypass.

### 4.1. Polκ on PCNA in the Act of Catalysis

In 2021, Lancey et al. [96] reported the high-resolution structures of full-length *Hs* Polκ in association with p/t DNA and unmodified (3.4 Å) or ubiquitinated (6.4 Å) PCNA, providing the first view of a Y-family TLS polymerase in association with PCNA during catalysis (Figure 5A,B). The Cryo-EM structures of the Polκ catalytic core, including palm, thumb, fingers, and PAD domains with p/t DNA in the active site, align well with the previous X-ray crystal structures [118], albeit DNA orientation below the PAD domain deviates from that in the crystal structure and the N-clasp is poorly resolved in Cryo-EM maps. In both structures, Polκ sits on the front face of PCNA and engages a single PCNA protomer through an internal PIP-box motif (PIP1) following the Polκ PAD domain (Figure 1 and Figure 5A). A short linker between Polκ PAD and PIP1 forms an α-helix (the ‘inverting helix’), which contributes 733 Å^2^ to the buried surface area of the PCNA/Polκ interface (Figure 5A, right). The C-terminal part of Polκ beyond the PIP1 motif, including the UBZ domains and the PIP2 motif, is invisible in Cryo-EM maps, suggesting this region is dynamic.

The structure of Polκ in complex with p/t DNA and ubiquitinated PCNA reveals a transient nature of PPIs between Polκ UBZs and PCNA-attached ubiquitin moieties [96]. While the UBZ domains are invisible, weak electron density is present on the back face of PCNA, corresponding to a ubiquitin moiety attached to each protomer at residue K164 (Figure 5B). The best-defined density is for ubiquitin attached to the PCNA protomer bound to Polκ, which is more accessible for interactions with Polκ UBZs. The authors demonstrated that the synthetic activity of the Polκ PIP1 mutant is severely impaired relative to wild-type Polκ in the presence of unmodified PCNA, but is only moderately reduced in the presence of ubiquitinated PCNA. Consistent with the previous functional studies [53,54], this result implies that the internal PIP-box serves as the major tether of Polκ to PCNA, while the UBZ domains supply accessory contacts, partially rescuing Polκ activity when the PIP-box interaction is impaired.

The most striking feature of the Polκ holoenzyme bound to PCNA and p/t DNA is its tilted orientation relative to the clamp, such that DNA exits the catalytic core at a 47° angle relative to the PCNA axis (Figure 5C) [96]. As a result, DNA that extends beyond the core bends by 30° to thread through the PCNA ring and avoid clashing with its inner rim. While the role of such DNA bending remains unclear, the authors suggest it may facilitate the assembly of PCNA ‘tool belts’ [47] whereby several DNA processing enzymes can engage the clamp at the same time and handover DNA without dissociation. An example of such a ‘tool belt’ is provided by the Cryo-EM structure of *Hs* Polδ and Flap endonuclease 1 (FEN1) bound to different protomers of the same PCNA ring [88]. The tilted orientation of Polκ relative to PCNA observed by Cryo-EM may serve to shift Polκ away and make space on the clamp to accommodate another DNA polymerase, as illustrated by modeling Polκ and Polδ onto a single PCNA molecule (Figure 5D).

### 4.2. Polζ Holoenzyme and Rev1-Polζ Assembly—A View of the TLS Mutasome

The first high-resolution Cryo-EM structures of the *Sc* Polζ holoenzyme on p/t DNA [97,98,99] reveal a pentameric “daisy-chain” or ring of Rev3, Pol31, Pol32, and two copies of Rev7 (Rev7_A_ and Rev7_B_) in a dimer (Figure 6A). In the Polζ structure, the catalytic subunit Rev3 alone interacts with DNA, and forms contacts with all other subunits but Pol32, while the accessory subunits arrange into a regulatory module facing away from DNA. Rev3 utilizes its C-terminal CysB domain to recruit the Pol31/Pol32 module via PPIs with Pol31, and binds a dimer of Rev7_A_ and Rev7_B_ subunits with its RBM1 and RBM2 motifs. The Rev3 segment connecting RBM1 and RBM2 forms additional contacts with Rev7 protomers and bridges the Rev7 dimer with the palm and thumb domains of the Rev3 catalytic core. Rev7_B_ interacts with the Pol31/Pol32 module near the interface of Pol31 and the N-terminal domain of Pol32 (Pol32_N_) closing the Polζ ring. The pentameric ring structure of Polζ is unique among the B-family polymerases in that the accessory subunits of Polζ are organized in a more rigid manner [86,87,88,89]. While *Sc* Polδ adopts a two-lobed structure comprising the globular catalytic subunit Pol3 and a loosely attached regulatory Pol31/Pol32 _N_ module [86], Pol31 and Pol32_N_ within *Sc* Polζ are stabilized by extensive contacts with the CysB and exonuclease domains of Rev3 and with Rev7_B_ (Figure 6A). The Pol32 C-terminal region (Pol32_C_) containing a terminal PIP-box motif is disordered. However, given the Pol32 orientation in the Pol ζ structure, Pol32_C_ points outwards and could extend to bind PCNA (Figure 6A).

Within the *Sc* Polζ holoenzyme, Rev7_A_ and Rev7_B_ form an unexpected head-to-tail dimer, not seen before in other HORMA proteins (Figure 6B, top). In this dimer, the αC helices of Rev7_A_ and Rev7_B_ are spaced far apart, with the new interface formed between the αC region of Rev7_A_ and the C-terminal safety-belt region of Rev7_B_ (Figure 6B, top). In contrast, *Hs* Rev7, alone [95] or within the shieldin complex [149] acting in DSB repair, forms a symmetric head-to-head dimer via the canonical HORMA interface centered at the helix αC (Figure 6B, bottom). Malik et al. [97] noted that the entire Rev7 interacting region of Rev3 within *Sc* Polζ “weave a path” through the two Rev7 subunits, making extensive contacts to both protomers and likely forcing them into an unusual head-to-tail dimer. This view is supported by a biophysical study of *Sc* Rev7 revealing that, unlike its human counterpart, the isolated *Sc* Rev7 lacks the ability to dimerize in solution, which is a property acquired later in evolution [94]. Interestingly, only the Rev7_B_ subunit within *Sc* Polζ can accommodate Rev1-CT, while the respective site on Rev7_A_ is occluded, suggesting *Sc* Polζ binds Rev1 in 1:1 stoichiometry.

In 2024, Malik et al. [100] reported a Cryo-EM structure (3.5 Å) of the *Sc* Rev1/Polζ holocomplex during p/t DNA extension by the Rev3 subunit of Polζ. The ring-like architecture of *Sc* Polζ is well preserved in this complex, while only three regions of a single Rev1 protein bound to Polζ are resolved, including Rev1-CT, Rev1-BRCT, and a Rev1-M1 α-helix preceding the BRCT domain (Figure 6C). The Rev1 catalytic core and the following region, including UBM domains (Figure 1), were flexible and invisible in the Cryo-EM map. As expected, Rev1-CT uses one face to bind the Rev7_B_ subunit of Polζ, while also making contacts with Pol32_N_ and a Rev3 segment connecting the two RBMs, and leaves the opposite face open for PPIs with the Y-family TLS polymerases (Figure 6C). Remarkably, the N-terminal Rev1-M1 α-helix and the following Rev1-BRCT domain form extensive contacts with the NTD-palm linker in the Rev3 catalytic domain, and with the primer and template strands of DNA (Figure 6C, right), which is consistent with previous observations [59]. This module is located similarly to the PAD domain in the Y-family TLS polymerases, tightening their grip on DNA. Furthermore, modeling of PCNA onto the *Sc* Rev1/Polζ structure reveals that Rev1-BRCT is primed for binding to the IDCL region of PCNA (Figure 6C), which is in line with NMR studies of the *Sc* Rev1-BRCT/PCNA PPIs [58]. Overall, the structure of the *Sc* Rev1/Polζ complex has uncovered the architecture of this essential module of the TLS mutasome and provided insights into the cooperation of TLS polymerases during DNA lesion bypass.

## 5. Concluding Remarks

Over the past two decades, a wealth of structural information has been amassed unveiling the mechanisms of catalysis and the interaction network of TLS DNA polymerases, which complements ample functional data available for these enzymes. However, until recently the mechanistic understanding of how the TLS polymerases assemble into multiprotein complexes and cooperate within supramolecular assemblies has markedly lagged behind. The recent high-resolution Cryo-EM structures of the multisubunit replicative [86,87,88,89] and TLS [96,97,98,99,100] DNA polymerases and their higher-order assemblies have been a remarkable feat in clarifying their activity on damaged and undamaged DNA. For example, the structural studies of *Hs* Polκ [96] and Polδ [87,88] holoenzymes on a sliding clamp PCNA have provided new insights into PCNA ‘tool belts’ [47], which facilitate DNA transactions between polymerases attached to the same clamp. The structures of the *Sc* Polζ holoenzyme and Rev1/Polζ holocomplex [96,97,98,99,100] unraveled the mechanism of p/t extension by Polζ and provided the first glimpse of the TLS mutasome in action. In addition, the *Sc* Polζ and Rev1/Polζ structures have elucidated the functional significance of already-characterized PPIs and described new interfaces and interactions. Because of their involvement in the replicative bypass of DNA adducts formed by genotoxic chemotherapeutics, which promotes drug resistance in cancers [25,26,27,28,29,30,31], TLS DNA polymerases have emerged as promising anti-cancer drug targets [32,33,34,35,36]. To date, efforts to develop small-molecule TLS inhibitors have mainly focused on targeting key PPIs within the TLS mutasome mediated by Rev1-CT and Rev7 [150,151,152,153,154,155,156,157]. The newly characterized TLS interfaces, therefore, extend the range of targets for TLS inhibition, providing new opportunities for anti-cancer drug design.

## Data Availability

No new data were created or analyzed in this study. Data sharing is not applicable to this article.
